# Antibacterial activity of 3-methylbenzo[d]thiazol-methylquinolinium derivatives and study of their action mechanism

**DOI:** 10.1080/14756366.2018.1465055

**Published:** 2018-05-03

**Authors:** Ning Sun, Ruo-Lan Du, Yuan-Yuan Zheng, Qi Guo, Sen-Yuan Cai, Zhi-Hua Liu, Zhi-Yuan Fang, Wen-Chang Yuan, Ting Liu, Xiao-Mei Li, Yu-Jing Lu, Kwok-Yin Wong

**Affiliations:** a The Fifth Affiliated Hospital of Guangzhou Medical University, Guangzhou, P.R. China;; b Department of Applied Biology and Chemical Technology and State Key Laboratory of Chirosciences, The Hong Kong Polytechnic University, Hung Hom, Kowloon, Hong Kong, P.R. China;; c Institute of Natural Medicine and Green Chemistry, School of Chemical Engineering and Light Industry, Guangdong University of Technology, Guangzhou, P.R. China;; d State Key Laboratory of Ophthalmology, Zhongshan Ophthalmic Center, Sun Yat-sen University, Guangzhou, P.R. China;; e Goldenpomelo Biotechnology Co. Ltd, Meizhou514021, P.R. China

**Keywords:** Bacterial resistance, antibacterial activity, 3-methylbenzo[d]thiazol-methylquinolinium derivatives, cell division, FtsZ inhibition

## Abstract

The increasing incidence of multidrug resistant bacterial infection renders an urgent need for the development of new antibiotics. To develop small molecules disturbing FtsZ activity has been recognized as promising approach to search for antibacterial of high potency systematically. Herein, a series of novel quinolinium derivatives were synthesized and their antibacterial activities were investigated. The compounds show strong antibacterial activities against different bacteria strains including MRSA, VRE and NDM-1 *Escherichia coli*. Among these derivatives, a compound bearing a 4-fluorophenyl group (**A2**) exhibited a superior antibacterial activity and its MICs to the drug-resistant strains are found lower than those of methicillin and vancomycin. The biological results suggest that these quinolinium derivatives can disrupt the GTPase activity and dynamic assembly of FtsZ, and thus inhibit bacterial cell division and then cause bacterial cell death. These compounds deserve further evaluation for the development of new antibacterial agents targeting FtsZ.

## Introduction

Antimicrobial resistance is one of the major actual health plague. Due to the overuse and abuse of antibacterial drugs, bacteria develop resistance to conventional antibiotics at an alarming speed, and the treatment of antibiotic-resistant bacterial infections becomes more and more difficult^1^. Methicillin-resistant *Staphylococcus auerus* (MRSA) is a typical example of Gram-positive bacteria which have already shown resistance to the wildly prescribed antibiotics including methicillin as well as other β-lactam antibiotics, such as oxacillin and nafcillin. MRSA is responsible for many illnesses, ranging from skin infections to pneumonia. In 2011, the CDC estimated there were about 11,285 MRSA related deaths in United Stated^2^. This situation is also critical in Gram-negative bacteria infections. The WHO has released a list of the drug-resistant bacteria which new antibiotics are desperately needed. In this list, carbapenem resistant Gram-negative organisms are in the critical priority^3^. For example, the recently emerging New Delhi metallo-β-lactamase 1 (NDM-1) superbugs has made almost of the first-line clinical antibiotics ineffective^4^. Infections by antibiotic-resistant bacteria lead to high morbidity and mortality rates, however, there are limited treatment options for these infections to-date. There is an urgent need for the development of new antibacterial agents with innovative mechanisms of action to against the multidrug-resistant bacteria^5^.

Bacterial cell division is an essential process that has not yet been targeted by clinically approved antibiotics and thus it is a very important research area for antibacterial discovery. Bacterial cell division is believed to be critical in new antibiotic development because it is an essential process for bacterial survival and the bacterial divisome possesses a complex set of biochemical machinery that contains many proteins. The most important division proteins are widely conserved among bacterial pathogens and they are almost absent in eukaryotic cells^6^. Among these proteins, filamentous temperature sensitive protein Z (FtsZ) plays a critical role in cell division process. To initiate cell division, FtsZ assembles into protofilaments in a GTP dependent manner and forms a ring-like structure (Z-ring) at the division site^7^^,^^8^. Z-ring functions as a scaffold for the assembly of other cell division proteins to form bacterial divisome. Although the composition and the interdependency of divisome members may vary among different species, most bacteria depend on FtsZ as the central pacemaker protein^9^. Therefore, FtsZ is an attractive target for the development of novel antimicrobials.

**Figure 1.**
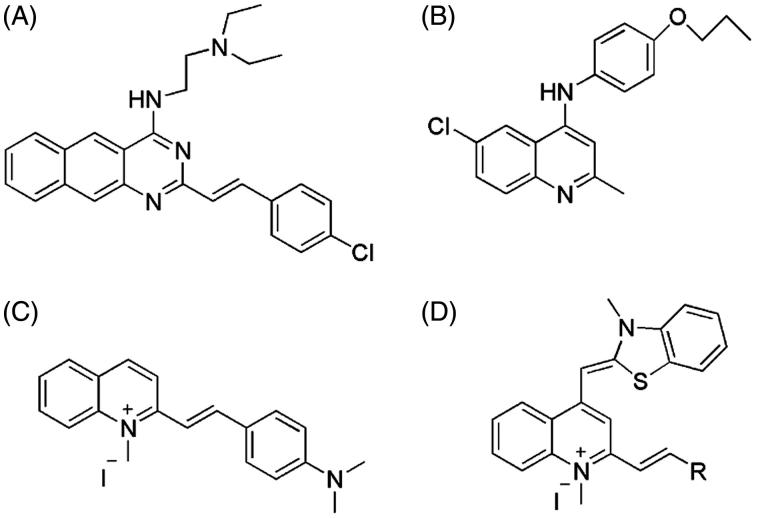
Structures of zantrin Z3, quinoline derivative, quinolinium derivative, and 3-methylbenzo[d]thiazol-methylquinolinium derivatives.

Over the past decade, only few inhibitors of FtsZ have been reported showing the potency of disrupting FtsZ function and causing filamentation in bacteria^10–12^. However, these examples reveal that FtsZ targeting compounds inhibit bacterial growth through disrupting the dynamic polymerization and/or GTP hydrolysis of FtsZ. Among the FtsZ inhibitors, zantrin Z3 (Figure 1(A)) and its analogs which contain a benzo[g]quinazoline core can effectively inhibit the GTPase activity of FtsZ and display a broad-spectrum and modest antibacterial activity against a panel of bacteria^13^^,^^14^. Further SAR study revealed that replacing benzo[g]quinazoline by a smaller quinazoline, these molecules retain inhibitory activity on the FtsZ protein^14^. A quinoline derivative (Figure 1(B)) were reported to inhibited the growth of *Mycobacterium tuberculosis* through disrupting the polymerization of *Mtb*FtsZ^15^. In addition, recent studies revealed that quinolinium derivatives (Figure 1(C)) have a potent antibacterial activity against drug resistant strains, including MRSA and Vancomycin-Resistant *E. faecalis*^16^^,^^17^. Based on our previous findings and experience on searching for novel potent anti-FtsZ agents through molecular design and synthesis^18–24^, we herein reported a new series of compounds (**A1**–**A16**) with a general molecular scaffold of 3-methylbenzo[d]thiazol-methylquinolinium (Figure 1(D)), in which we systemically varying the substituent groups linked at the ortho-position of 1-methylquinolinium core and investigated their antimicrobial activity with respect to different styryl substituents and mode of action targeting FtsZ.

## Materials and methods

### Chemistry

#### General experimental

Melting points (m.p.) were determined using a SRS Opti Mel automated melting point instrument without correction. ^1^H and 13C NMR spectra were recorded using TMS as the internal standard in DMSO-d_6_ with a Bruker BioSpin GmbH spectrometer at 400 and 100 MHz, respectively. Mass spectra (MS) were recorded on Bruker amaZon SL mass spectrometer with an ESI or ACPI mass selective detector. Reactions progress and compounds were checked by TLC with Merck silica gel 60F-254 glass plates. All chemicals were purchased from commercial sources unless otherwise specified, and all the solvents were analytical grade. The purities of synthesized compounds were confirmed by HPLC with a dual pump Shimadzu LC-20A system equipped with a photo-diode array detector and a C18 column (250 × 4.6 mm, 5 μM YMC) and eluted with acetonitrile/water (47:53) containing 0.5% acetic acid at flow rate of 1.0 ml/min.

#### Synthesis of 4-chloro-1,2-dimethylquinolin-1-ium iodide (I1)

Iodomethane (0.42 ml, 6.74 mmol) was added into the solution of 4-Chloro-2-methylquinoline (0.2 g, 1.12 mmol) in sulfolane (10 ml) was added. The reaction mixture was stirred at 50 °C for 20 h, cooled and anhydrous ether is added after the shock, suction filtration, the solid was washed with anhydrous diethyl ether, dried in vacuum to give of **I1** (0.343 g, 95%): mp: 245–247 °C. ^1^H NMR (400 MHz, DMSO-d_6_): δ 8.90 (s, 1H), 8.67 (d, *J* = 9.0 Hz, 1H), 8.26 (ddd, *J* = 8.7, 7.1, 1.4 Hz, 1H), 8.12 (t, *J* = 7.6 Hz, 1H), 8.05 (t, *J* = 7.5 Hz, 1H), 4.43 (s, 3H), 2.99 (d, *J* = 6.2 Hz, 3H). ESI-MS: *m/z* 192.1 [M − I]^+^.

#### Synthesis of 1, 2-dimethyl–benzo[d]thiazol-1-ium iodide (I2)

A mixture of 2-methylbenzo[d]thiazole (0.25 g, 1.68 mmol), iodomethane (0.63 ml, 10.08 mmol) and anhydrous ethanol (10 ml) was stirred at reflux temperature for 15 h. After cooling, the mixture was dried over anhydrous ethanol and chloroform oscillating suction filtered. The precipitate was washed with chloroform and a small amount of ethanol, then vacuum dried to give **I2** (0.447 g, 91.7%): mp: 232–235 °C. ^1^H NMR (400 MHz, DMSO-d_6_): δ 8.44 (d, *J* = 8.1 Hz, 1H), 8.30 (d, *J* = 8.4 Hz, 1H), 7.90 (t, *J* = 7.8 Hz, 1H), 7.81 (t, *J* = 7.7 Hz, 1H), 4.20 (s, 3H), 3.17 (s, 3H). ESI-MS: *m/z* 164.4 [M − I]^+^.

#### Synthesis of (Z)-1,2-dimethyl-4-((3-methylbenzo[d]thiazol-2(3H)-ylidene)methyl) quinolin-1-ium iodide (I3)

**I1** (0.5 g, 1.60 mmol), **I2** (0.5 g, 1.75 mmol) and aqueous sodium bicarbonate solution (0.5 mol/l, 2 ml) were mixed with 10 ml methanol, and stirred at room temperature. After 1 h, 4 ml saturated KI solution was added to the reaction solution. After stirred another 15 min, **I3** was obtained by washing with water and acetone, and dried in vacuum (0.475 g, 92%): mp: 268–271 °C. ^1^H NMR (400 MHz, DMSO-d_6_): δ 8.77 (d, *J* = 8.3 Hz, 1H), 8.18 (d, *J* = 8.7 Hz, 1H), 8.02–7.96 (m, 2H), 7.74 (d, *J* = 8.2 Hz, 2H), 7.59 (t, *J* = 7.7 Hz, 1H), 7.39 (t, *J* = 7.5 Hz, 1H), 7.33 (s, 1H), 6.84 (s, 1H), 4.06 (s, 3H), 3.98 (s, 3H), 2.87 (s, 3H). ESI-MS: *m/z* 319.0 [M − I]^+^.

#### General procedure for the synthesis of 3-methylbenzo[d]thiazol-methylquinolinium derivatives (A1-A16)

A mixture of **I3** (0.072 g, 0.16 mmol), 4-methylpiperidine (0.5 ml), *n*-butanol (10 ml) and selected aldehyde (0.32 mmol) were mixed and stirred at reflux temperature for 3 h. After the mixture was cooled down and filtered by suction, the solid was washed with *n*-butanol and purified by using column chromatography to obtain the pure target compounds **A1**–**A16**.

*2-((E)-4-chlorostyryl)-1-methyl-4-((E)-(3-methylbenzo[d]thiazol-2(3H)-ylidene) methyl)quinolin-1-ium iodide (****A1****).* Purple solid, yield 85%; mp 297–301 °C; ^1^H NMR (400 MHz, DMSO*-*d_6_): δ 8.72 (d, *J* = 8.4 Hz, 1H), 8.24 (d, *J* = 8.68 Hz, 1H), 8.08 (d, 1H), 8.02 (d, *J* = 7.4 Hz,3H), 7.84 (d, *J* = 4.5 Hz, 1H), 7.75 (m, *J* = 9.5 Hz, 3H), 7.65 (s, 1H), 7.56 (s, 3H), 7.45 (t, *J* = 8.3 Hz, 1H), 6.95 (s, 1H), 4.27 (s, 3H), 4.02 (s, 3H). 13C NMR (100 MHz, DMSO-d_6_): δ 159.31, 154.79, 148.40, 140.95, 139.64, 139.49, 133.58, 130.62, 129.43, 128.50, 126.83, 125.82, 124.67, 124.14, 123.91, 123.48, 123.17, 118.73, 113.10, 111.03, 87.33, 37.56, 34.09. ESI-MS: [M − I]^+^ (C_27_H_22_ClN_2_S^+^): *m/z* 441.0; HPLC retention time was 1.94 min.

*2-((E)-4-fluorostyryl)-1-methyl-4-((E)-(3-methylbenzo[d]thiazol-2(3H)-ylidene)methyl) quinolin-1-ium iodide (****A2****).* Rufous solid, yield 85%; mp 293–296 °C; ^1^H NMR (400 MHz, DMSO-d_6_): δ 8.69 (d, *J* = 8.4 Hz, 1H), 8.01 (dd, *J* = 29.3, 10.1 Hz, 4H), 7.94–7.89 (m, 1H), 7.70 (d, *J* = 7.6 Hz, 1H), 7.63 (dd, *J* = 19.6, 11.2 Hz, 3H), 7.54 (d, *J* = 14.5 Hz, 1H), 7.44 (s, 1H), 7.34 (dd, *J* = 19.2, 8.3 Hz, 3H), 6.79 (s, 1H), 4.07 (s, 3H), 3.92 (s, 3H). 13C NMR (100 MHz, DMSO-d_6_): δ 159.83, 152.07, 148.00, 140.82, 139.79, 139.32, 133.66, 132.22, 131.24, 131.16, 128.49, 126.90, 125.61, 124.66, 124.27, 123.85, 123.42, 121.96, 118.89, 116.47, 116.25, 113.09, 108.23, 88.29, 38.53, 34.13. ESI-MS: [M − I]^+^ (C_27_H_22_FN_2_S^+^): *m/z* 425.0; HPLC retention time was 3.63 min.

*2-((E)-4-bromostyryl)-1-methyl-4-((E)-(3-methylbenzo[d]thiazol-2(3H)-ylidene) methyl)quinolin-1-ium iodide (****A3****).* Rufous solid, yield 87%; Mp 307–309 °C; ^1^HNMR (400 MHz, DMSO-d_6_): δ 8.76 (d, *J* = 8.2 Hz, 1H), 8.18 (d, *J* = 8.2 Hz, 1H), 8.05 (d, *J* = 7.6 Hz, 1H), 8.00 (d, *J* = 7.3 Hz, 1H), 7.90 (d, *J* = 7.8 Hz, 2H), 7.86 – 7.70 (m, 5H), 7.63 (d, *J* = 19.0 Hz, 3H), 7.44–7.37 (m, 1H), 6.92 (s, 1H), 4.12 (d, *J* = 30.4 Hz, 3H), 4.01 (s, 3H). 13C NMR (100 MHz, DMSO-d_6_): δ 159.57, 154.63, 148.40, 140.95, 139.64, 139.49, 133.58, 130.62, 129.43, 128.50, 126.83, 125.82, 124.67, 124.14, 123.91, 123.48, 123.17, 118.73, 113.10, 111.03, 87.33, 37.56, 34.09. ESI-MS: [M − I]^+^ (C_27_H_22_BrN_2_S^+^): *m/z* 486.9; HPLC retention time was 3.52 min.

*2-((E)-2,4-dichlorostyryl)-1-methyl-4-((E)-(3-methylbenzo[d]thiazol-2(3H)-ylidene)methyl)quinolin-1-ium iodide (****A4****).* Purple solid, yield 87%; Mp 271–275 °C; ^1^H NMR (400 MHz, DMSO-d_6_): δ 8.74 (d, *J* = 7.8 Hz, 1H), 8.18 (d, *J* = 8.7 Hz, 1H), 8.10 (d, *J* = 8.5 Hz, 1H), 7.96 (d, *J* = 7.0 Hz, 2H), 7.85 (d, *J* = 15.8 Hz, 1H), 7.73 (t, *J* = 17.6 Hz, 4H), 7.59 (t, *J* = 10.7 Hz, 2H), 7.52 (s, 1H), 7.39 (d, *J* = 7.2 Hz, 1H), 6.91 (s, 1H), 4.11 (s, 3H), 3.98 (s, 3H). 13C NMR (100 MHz, DMSO-d_6_): δ 159.90, 151.68, 148.25, 140.85, 139.37, 135.67, 134.83, 134.75, 133.87, 132.23, 130.11, 129.89, 128.67, 128.48, 127.11, 125.98, 125.67, 124.94, 123.97, 123.19, 118.98, 113.33, 108.68, 88.78, 38.68, 34.31. ESI-MS: [M − I]^+^ (C_27_H_21_Cl_2_N_2_S^+^): *m/z* 475.0; HPLC retention time was 4.29 min.

*1-methyl-4-((E)-(3-methylbenzo[d]thiazol-2(3H)-ylidene)methyl)-2-((E)-4-methyl styryl)quinolin-1-ium iodide (****A5****).* Red solid, yield 85%; mp 275–278 °C; ^1^H NMR (400 MHz, DMSO-d_6_): δ 8.77 (d, *J* = 8.3 Hz, 1H), 8.19 (d, *J*= 8.8 Hz, 1H), 8.07 (d, *J*= 7.8 Hz, 1H), 8.00 (t, *J*= 7.9 Hz, 1H), 7.85 (d, *J* = 7.9 Hz, 2H), 7.75 (dd, *J* = 9.7, 5.6 Hz, 3H), 7.63 (dt, *J* = 15.6, 9.9 Hz, 3H), 7.43–7.34 (m, 3H), 6.92 (s, 1H), 4.17 (s, 3H), 4.00 (d, *J* = 8.1 Hz, 3H), 2.41 (s, 3H). ^13^C NMR (100 MHz, DMSO-d_6_): δ 159.92, 152.60, 148.26, 141.32, 141.05, 140.79, 139.52, 133.74, 132.99, 130.03, 128.95, 128.55, 126.97, 125.69, 124.69, 124.27, 124.01, 123.48, 121.10, 119.03, 113.14, 108.44, 88.27, 38.53, 34.12, 21.57. ESI-MS: [M − I]^+^ (C_28_H_25_N_2_S^+^): *m/z* 421.2; HPLC retention time was 3.38 min.

*2-((E)-4-(dimethylamino)styryl)-1-methyl-4-((E)-(3-methylbenzo[d]thiazol-2(3H)-y lidene)methyl)quinolin-1-ium iodide (****A6****).* Purple solid, yield 89%; mp 301–304 °C; ^1^H NMR (400 MHz, DMSO-d_6_): δ 8.70 (d, *J* = 8.3 Hz, 1H), 8.13 (d, *J* = 8.8 Hz, 1H), 8.03 (d, *J* = 7.7 Hz, 1H), 7.97–7.92 (m, 1H), 7.79 (d, *J* = 8.9 Hz, 2H), 7.71 (d, *J* = 7.8 Hz, 1H), 7.69–7.64 (m, 2H), 7.63 (s, 1H), 7.56 (t, *J* = 7.8 Hz, 1H), 7.43 (d, *J* = 15.7 Hz, 1H), 7.36 (t, *J* = 7.6 Hz, 1H), 6.79 (d, *J* = 9.1 Hz, 3H), 4.13 (s, 3H), 3.94 (s, 3H), 3.05 (s, 6H). 13C NMR (100 MHz, DMSO-d_6_): δ 158.77, 153.24, 152.30, 147.53, 142.83, 141.10, 139.57, 133.43, 130.97, 128.40, 126.63, 125.57, 124.30, 124.05, 123.92, 123.38, 123.11, 118.93, 115.10, 112.74, 112.16, 108.03, 87.58, 38.28, 33.91. ESI-MS: [M − I]^+^ (C_29_H_28_N_3_S^+^): *m/z* 450.1; HPLC retention time was 5.44 min.

*1-methyl-4-((E)-(3-methylbenzo[d]thiazol-2(3H)-ylidene)methyl)-2-((E)-4-(methyl thio)styryl)quinolin-1-ium iodide (****A7****).* Rufous solid, yield 90%; mp 293–295 °C; ^1^H NMR (400 MHz, DMSO-d_6_): δ 8.73 (d, *J* = 7.8 Hz, 1H), 8.14 (d, *J* = 8.4 Hz, 1H), 8.06–8.02 (m, 1H), 8.00–7.95 (m, 1H), 7.87 (d, *J* = 8.5 Hz, 2H), 7.76–7.68 (m, 3H), 7.64 (s, 1H), 7.61–7.55 (m, 2H), 7.42–7.35 (m, 3H), 6.87(s, 1H), 4.13(s, 3H), 3.97 (d, *J* = 3.7 Hz, 3H), 2.56 (s, 3H). 13C NMR (100 MHz, DMSO-d_6_): δ 159.81, 152.50, 148.16, 142.08, 141.04, 140.86, 139.51, 133.71, 132.08, 129.41, 128.54, 126.94, 126.07, 125.67, 124.68, 124.26, 123.98, 123.44, 120.95, 119.01, 113.11, 108.42, 38.53, 34.11, 14.77. ESI-MS: [M − I]^+^ (C_28_H_25_N_2_S_2_^+^): *m/z* 453.0; HPLC retention time was 3.45 min.

*2-((E)-4-methoxystyryl)-1-methyl-4-((E)-(3-methylbenzo[d]thiazol-2(3H)-ylidene) methyl)quinolin-1-ium iodide (****A8****).* Brown solid, yield 86%; mp 263–267 °C; ^1^H NMR (400 MHz, DMSO-d_6_): δ 8.73 (d, *J* = 8.4 Hz, 1H), 8.08 (dd, *J* = 14.8, 8.3 Hz, 2H), 7.99–7.89 (m, 3H), 7.73 (t, *J* = 7.6 Hz, 1H), 7.66 (d, *J* = 8.3 Hz, 1H), 7.63–7.56 (m, 3H), 7.51 (s, 1H), 7.40 (t, *J* = 7.5 Hz, 1H), 7.09 (d, *J* = 8.5 Hz, 2H), 6.82 (s, 1H), 4.11 (s, 3H), 3.96 (s, 3H), 3.91 (s, 3H). ^13^C NMR (100 MHz, DMSO-d_6_): δ 160.41, 158.29, 151.39, 146.68, 140.01, 139.75, 138.21, 132.41, 129.64, 127.29, 127.12, 125.65, 124.43, 123.36, 123.04, 122.70, 122.25, 118.06, 117.75, 113.67, 111.81, 106.95, 86.87, 54.80, 37.30, 32.91. ESI-MS: [M − I]^+^ (C_28_H_25_N_2_OS^+^): *m/z* 437.0; HPLC retention time was 2.95 min.

*2-((E)-3-methoxystyryl)-1-methyl-4-((E)-(3-methylbenzo[d]thiazol-2(3H)-ylidene) methyl)quinolin-1-ium iodide (****A9****).* Rufous solid, yield 87%; mp 252–256 °C; ^1^H NMR (400 MHz, DMSO-d_6_): δ 8.50 (d, *J* = 8.5 Hz, 1H), 7.83–7.73 (m, 3H), 7.70 (d, *J* = 7.7 Hz, 1H), 7.56 (dd, *J* = 15.5, 11.8 Hz, 2H), 7.39 (dd, *J* = 16.2, 9.3 Hz, 3H), 7.31 (d, *J* = 8.2 Hz, 1H), 7.24 (t, *J* = 7.5 Hz, 1H), 7.09 (s, 1H), 6.99 (t, *J* = 7.4 Hz, 1H), 6.92 (d, *J* = 8.3 Hz, 1H), 6.52 (s, 1H), 3.81 (d, *J* = 12.7 Hz, 3H), 3.74 (s, 3H), 3.65 (s, 3H). ^13^C NMR (100 MHz, DMSO-d_6_): δ 158.57, 157.74, 152.11, 147.18, 140.32, 138.85, 135.87, 133.43, 132.33, 128.64, 128.40, 126.69, 125.29, 124.49, 123.62, 123.54, 123.47, 122.65, 121.28, 121.09, 118.61, 112.79, 111.99, 107.86, 87.84, 56.28, 38.22, 34.02. ESI-MS: [M − I]^+^ (C_28_H_25_N_2_OS^+^): *m/z* 437.0; HPLC retention time was 3.14 min.

*2-((E)-3,4-dimethoxystyryl)-1-methyl-4-((E)-(3-methylbenzo[d]thiazol-2(3H)-ylidene)methyl)quinolin-1-ium iodide (****A10****).* Brown solid, yield 89%; mp 266–270 °C; ^1^H NMR (400 MHz, DMSO-d_6_): δ 8.73 (d, *J* = 8.2 Hz, 1H), 8.14 (s, 1H), 8.05 (d, *J* = 7.8 Hz, 1H), 7.99–7.95 (m, 1H), 7.91 (d, *J* = 7.3 Hz, 1H), 7.69 (s, 1H), 7.62 (d, *J* = 6.7 Hz, 2H), 7.58 (s, 3H), 7.47 (d, *J* = 8.1 Hz, 1H), 7.39 (d, *J* = 7.6 Hz, 2H), 7.07 (d, *J* = 8.2 Hz, 1H), 6.85 (s, 1H), 6.65 (s, 1H), 4.16 (s, 3H), 3.97 (s, 3H), 3.91 (s, 3H), 3.85 (s, 3H). ^13^C NMR (100 MHz, DMSO-d_6_): δ 158.57, 157.74, 152.11, 147.13, 140.32, 138.85, 135.87, 133.43, 132.33, 128.64, 128.40, 126.69, 125.29, 124.49, 123.62, 123.54, 123.47, 122.65, 122.41, 87.70, 56.28, 37.96, 33.94. ESI-MS: [M − I]^+^ (C_29_H_27_N_2_O_2_S^+^): *m/z* 467.0; HPLC retention time was 5.72 min.

*1-methyl-4-((E)-(3-methylbenzo[d]thiazol-2(3H)-ylidene)methyl)-2-((E)-2-(pyridine-3-yl)vinyl)quinolin-1-ium iodide (****A11****).* Rufous solid, yield 88%; mp 288–291 °C; ^1^H NMR (400 MHz, DMSO-d_6_): δ 9.10 (s, 1H), 8.77 (s, 1H), 8.66 (d, *J* = 3.7 Hz, 1H), 8.39 (d, *J* = 7.3 Hz, 1H), 8.17 (s, 1H), 8.06 (d, *J* = 7.5 Hz, 1H), 7.99 (s, 1H), 7.91 (d, *J* = 15.9 Hz, 1H), 7.74 (s, 2H), 7.67 (d, *J* = 18.3 Hz, 1H), 7.65–7.52 (m, 3H), 7.41 (s, 1H), 6.91 (s, 1H), 4.16 (s, 3H), 4.00 (s, 3H). ^13^C NMR (100 MHz, DMSO-d_6_): δ 160.23, 151.88, 151.12, 150.40, 148.29, 140.98, 139.45, 137.63, 135.15, 133.81, 131.47, 128.59, 127.05, 125.70, 124.83, 124.41, 124.37, 124.29, 123.98, 123.45, 119.01, 113.27, 108.47, 88.58, 38.62, 34.22. ESI-MS: [M − I]^+^ (C_26_H_22_N_3_S^+^): *m/z* 408.0; HPLC retention time was 1.97 min.

*2-((E)-2–(1H-indol-3-yl)vinyl)-1-methyl-4-((E)-(3-methylbenzo[d]thiazol-2(3H)-ylidene)methyl)quinolin-1-ium iodide (****A12****).* Rufous solid, yield 85%; mp 298–303 °C; ^1^H NMR (400 MHz, DMSO-d_6_): δ 11.97 (s, 1H), 8.62 (d, *J* = 8.4 Hz, 1H), 8.20 (s, 1H), 8.09 (d, *J* = 7.3 Hz, 1H), 8.03 (d, *J* = 8.8 Hz, 1H), 7.98 (d, *J* = 7.8 Hz, 1H), 7.88 (dd, *J* = 15.5, 5.5 Hz, 2H), 7.63 (t, *J* = 7.6 Hz, 1H), 7.51 (q, *J* = 8.4 Hz, 4H), 7.33–7.23 (m, 4H), 6.70 (d, *J* = 8.5 Hz, 1H), 4.07 (s, 3H), 3.83 (s, 3H). ^13^C NMR (100 MHz, DMSO-d_6_): δ 158.34, 153.65, 147.23, 140.96, 139.40, 136.84, 133.30, 128.29, 126.47, 125.49, 125.13, 124.14, 123.92, 123.80, 123.38, 123.26, 121.71, 120.70, 118.78, 114.21, 113.98, 113.05, 112.54, 107.63, 87.39, 38.20, 33.84. ESI-MS: [M − I]^+^ (C_29_H_24_N_3_S^+^): *m/z* 446.1; HPLC retention time was 2.81 min.

*1-methyl-4-((E)-(3-methylbenzo[d]thiazol-2(3H)-ylidene)methyl)-2-((E)-2-(naphth alen-2-yl)vinyl)quinolin-1-ium iodide (****A13****).* Black solid, yield 85%; mp 299–304 °C; ^1^H NMR (400 MHz, DMSO-d_6_): δ 8.78 (d, *J* = 8.3 Hz, 1H), 8.41 (s, 1H), 8.18 (t, *J* = 8.1 Hz, 2H), 8.11–8.04 (m, 2H), 8.01 (d, *J* = 4.9 Hz, 3H), 7.91 (s, 1H), 7.85 (s, 1H), 7.75 (t, *J* = 8.6 Hz, 2H), 7.67 (s, 1H), 7.65–7.56 (m, 3H), 7.40 (t, *J* = 7.4 Hz, 1H), 6.94 (d, *J* = 9.8 Hz, 1H), 4.20 (s, 3H), 3.99 (s, 3H). 13C NMR (100 MHz, DMSO-d_6_): δ 159.99, 152.42, 148.28, 141.24, 141.06, 139.55, 134.15, 133.77, 133.39, 133.34, 130.41, 129.05, 128.89, 128.56, 128.30, 127.90, 127.44, 126.99, 125.73, 124.72, 124.58, 124.32, 124.05, 123.52, 122.55, 119.06, 113.19, 108.54, 88.44, 38.63, 34.18. ESI-MS: [M − I]^+^ (C_31_H_25_N_2_S^+^): *m/z* 457.0; HPLC retention time was 6.90 min.

*1-methyl-4-((Z)-(3-methylbenzo[d]thiazol-2(3H)-ylidene)methyl)-2-((1E,3E)-4-phenylbuta-1,3-dien-1-yl)quinolin-1-ium iodide (****A14****).* Brown solid, yield 80%; mp 254–257 °C; ^1^H NMR (400 MHz, DMSO-d_6_): δ 8.73 (t, *J* = 10.7 Hz, 1H), 8.10 (dd, *J* = 18.2, 9.6 Hz, 2H), 7.97–7.92 (m, 1H), 7.74–7.68 (m, 2H), 7.67–7.61 (m, 2H), 7.60–7.51 (m, 3H), 7.45 (dd, *J* = 13.6, 5.9 Hz, 2H), 7.42–7.35 (m, 3H), 7.31 (d, *J* = 15.2 Hz, 2H), 6.83 (d, *J* = 9.2 Hz, 1H), 4.06 (d, *J* = 14.2 Hz, 3H), 3.97 (d, *J* = 7.1 Hz, 3H). ^13^C NMR (100 MHz, DMSO-d_6_): δ 159.64, 151.84, 147.88, 141.95, 141.06, 140.24, 139.50, 136.52, 133.65, 129.66, 129.53, 128.60, 128.54, 127.64, 126.90, 125.64, 125.09, 124.64, 124.24, 123.96, 123.48, 118.98, 113.11, 108.00, 88.23, 38.24, 34.12. ESI-MS: [M − I]^+^ (C_29_H_25_N_2_S^+^): *m/z* 433.2; HPLC retention time was 1.70 min.

*2-((1E,3E)-4–(4-fluorophenyl)buta-1,3-dien-1-yl)-1-methyl-4-((Z)-(3-methylbenzo [d]thiazol-2(3H)-ylidene)methyl)quinolin-1-ium iodide (****A15****).* Trovirens solid, yield 87%; mp 281–284 °C; ^1^H NMR (400 MHz, DMSO-d_6_): δ 8.72 (d, *J* = 8.3 Hz, 1H), 8.09 (t, *J* = 9.5 Hz, 2H), 7.98–7.93 (m, 1H), 7.70 (dd, *J* = 15.8, 7.2 Hz, 4H), 7.61–7.56 (m, 1H), 7.55–7.49 (m, 2H), 7.42–7.37 (m, 1H), 7.31 (t, *J* = 8.8 Hz, 4H), 7.27 (s, 1H), 6.85 (s, 1H), 4.08 (s, 3H), 3.97 (s, 3H). ^13^C NMR (100 MHz, DMSO-d_6_): δ 164.19, 161.73, 159.63, 151.82, 147.89, 141.86, 141.05, 139.49, 138.96, 133.64, 133.20, 133.17, 129.74, 129.66, 128.53, 128.44, 126.89, 125.63, 124.99, 124.62, 124.23, 123.96, 123.47, 118.93, 116.60, 116.38, 113.09, 108.01, 88.24, 38.23, 34.14. ESI-MS: [M − I]^+^ (C_29_H_24_FN_2_S^+^): *m/z* 451.0; HPLC retention time was 1.71 min.

*2-((1E,3E)-4–(4-(dimethylamino)phenyl)buta-1,3-dien-1-yl)-1-methyl-4-((Z)-(3-methylbenzo[d]thiazol-2(3H)-ylidene)methyl)quinolin-1-ium iodide (****A16****).* Atrovirens solid, yield 85%; mp: 289–294 °C; ^1^H NMR (400 MHz, DMSO-d_6_): δ 8.70 (d, *J* = 8.5 Hz, 1H), 8.12 (d, *J* = 8.9 Hz, 1H), 8.07 (d, *J* = 7.9 Hz, 1H), 7.95 (t, *J* = 7.8 Hz, 1H), 7.73–7.68 (m, 2H), 7.59 (dd, *J* = 13.5, 7.0 Hz, 3H), 7.49 (d, *J* = 8.7 Hz, 2H), 7.39 (t, *J* = 7.6 Hz, 1H), 7.17 (dd, *J* = 24.7, 13.8 Hz, 3H), 6.83–6.76 (m, 3H), 4.09 (d, *J* = 8.5 Hz, 3H), 3.96 (s, 3H), 3.00 (d, *J* = 11.7 Hz, 6H). ^13^C NMR (100 MHz, DMSO-d_6_): δ 158.75, 152.13, 151.49, 147.13, 143.68, 142.22, 140.98, 139.42, 133.35, 129.38, 128.39, 126.59, 125.49, 124.30, 124.08, 124.00, 123.81, 123.62, 123.41, 120.93, 118.77, 112.78, 112.53, 107.66, 87.68, 37.99, 33.94. ESI-MS: [M − I]^+^ (C_31_H_30_N_2_S^+^): *m/z* 476.1. HPLC retention time was 4.89 min.

### Antimicrobial susceptibility assays

Antimicrobial susceptibility tests were conducted in 96-well microplates using the broth microdilution procedure in accordance to the Clinical and Laboratory Standards Institute (CLSI) guidelines^25^. Cation-adjusted Mueller Hinton broth for all the *S. aureus* strains, including MRSA, or brain heart infusion broth for antibiotic-susceptible *E. faecium* ATCC 49624 and *E. faecalis* ATCC 29212, vancomycin-resistant *E. faecium* ATCC 700221 and *E. faecalis* ATCC 51575, or Mueller Hinton broth for the other strains were used in the assays. After incubation for 18 h at 37 °C, the absorbance at 600 nm (A_600_) was recorded using a microplate reader (Bio-Rad laboratory Ltd., UK) and the percentage of bacterial cell inhibition with respect to vehicles (1% DMSO) was calculated. The MIC was defined as the lowest compound concentration at which the growth of bacteria was inhibited by ≥90%. Three independent assays were performed for each test.

### Time-killing curve determination

A growing culture of *S. aureus* ATCC 29213 or *E. coli* ATCC25922 were diluted to approximately 10^5^ CFU mL^−1^ in volumes of Cation-adjusted Mueller Hinton broth or Mueller Hinton broth, respectively, containing various concentrations of tested compound. Cultures were incubated with shaking at 37 °C. At the appropriate time intervals, 100 μL samples were removed for serial dilution in 900 μL volumes of corresponding medium, and 100 μL volumes from three dilutions were spread on to MH agar. Cell counts (CFU ml^−1^) were enumerated after incubating the plates at 37 °C for 24 h.

### GTPase activity test

FtsZ protein in the biological tests was prepared as our previous report^21^. The GTPase activity of *Sa*FtsZ was measured in 96-well microplates using a phosphate assay Kit according to previous description^20^. FtsZ (3.5 μM) was preincubated with different concentrations of tested compounds in 20 mM Tris buffer (pH 7.4, 0.01%Triton X-100 to avoid compound aggregation) at room temperature. 5 mM of MgCl_2_ and 200 mM of KCl were added after 10 min incubation. Reactions were started with the addition of 500 mM GTP and incubated in a water bath at 37 °C. After 30 min, the reactions were quenched by adding 100 μL of Cytophos reagent for 10 min. Inorganic phosphate was quantified by measuring the absorbance at 650 nm with a microplate reader.

### Light scattering assay

The FtsZ polymerization was measured using 90° light scattering in a fluorescence spectrometer at 37 °C. Excitation and emission wavelengths were set at 600 nm with a slit width of 2.5 nm. FtsZ (5 μM) in 20 mM of Tris buffer (pH 7.4, containing 0.01%Triton X-100 to avoid compound aggregation) was placed in a fluorometer cuvette, and the polymerization reaction was started by consecutive additions of 20 mM KCl, 5 mM MgCl_2_, 1 mM GTP, and different concentrations of tested compound.

### Transmission electron microscopy (TEM)

FtsZ (13.5 μM) was incubated with different concentrations of tested compounds in 20 mM Tris buffer (pH 7.4) at room temperature. After 10 min incubation, 5 mM MgCl_2_, 50 mM KCl, and 1 mM GTP were added to the reaction mixtures and incubated at 37 °C for another 10 min. Then, 10 μL of the sample mixtures were dropped on a glow-discharged Formvar carbon-coated copper grid. The grids were subsequently subjected to negative staining using 10 μL of 0.5% phosphotungstic acid for 1 min, air-dried and digital images of the specimen were obtained from a transmission electron microscope (JEOL model JEM 2010).

### Bacterial morphology study

The *B. subtilis* 168 was grown in LB medium. The cultures at an A_600_ of 0.01 from an overnight culture were inoculated in the same medium containing different concentrations of the tested compound and grown at 37 °C for 4 h. The cells for morphology studies were harvested and resuspended in 100 μL of PBS buffer containing 0.25% agarose. 10 μL of the suspension mixture were then placed on a microscopic slide and the morphology of the bacterial cells was observed under a light phase-contrast microscope.

### Molecular modeling

The molecular modeling were performed using Discovery Studio 2016. The X-ray crystal structure of FtsZ was downloaded from the PDB database (PDB entry: 4DXD; resolution: 2.0 Å)^26^. Co-crystal ligands and water molecules were removed from the structure and the protein was prepared for docking using automated procedure of Discovery Studio. The structures of **A2** was sketched and minimized using the Discovery Studio molecule preparation tools. The automated docking study was carried out using DS-CDocker protocol in the Discovery Studio. The highest scoring poses were visually inspected.

### Cytotoxicity test of quinolinium derivatives

Selected quinolinium derivatives (**A2** and **A5**) were tested with renal epithelial cells (HK-2) and Mouse fibroblasts cells (L929) to determine the cytotoxicity of eukaryotic mammalian cells. Cells were re-suspended in complete cell culture medium and the concentration was adjusted at approximately 1 × 10^5^ cells mL^−1^. Cells seeded in the 96 wells microtitre plates for 24 h were used for the evaluation of the tested compounds. For the MTS assay, cells (5,000 per well) were seeded into 96-well plates. After treatment with compounds of different concentrations for 24h, cells were added with MTS at a final concentration of 0.3 mg/mL, followed by incubation for another 2h. The optical density (OD) of each well was determined at 490 nm (background subtraction at 690 nm) by a SpectraMax 340 microplate reader. The growth inhibitory ratio was calculated as follows: Growth inhibitory ratio = (A_control_ − A_sample_)/A_control_ (where A is the OD value per well).

## Results and discussions

### Synthesis of target compounds

**Scheme 1.**
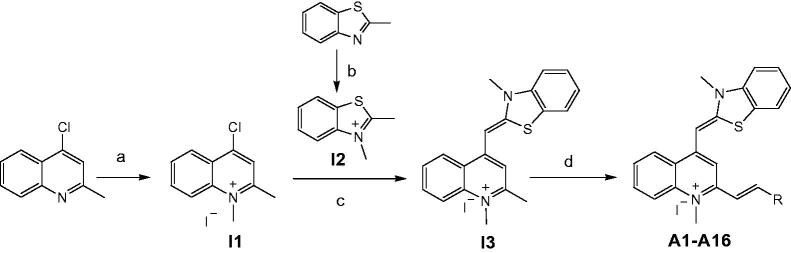
Synthesis route of 3-methylbenzo[d]thiazol-methylquinolinium derivatives. Reagents and conditions: (a) iodomethane, tetramethylene sulfone, 50 °C, reflux; (b) iodomethane, absolute ethanol, 80 °C, reflux; (c) NaHCO_3_, methanol, room temperature; (d) aromatic aldehydes, 4-methylpiperidine, *n*-butanol, 135 °C, reflux.

In the present study, compounds **A1**–**A16** listed in Table 1 were synthesized from 4-chloro-2-methylquinoline as outlined in Scheme 1. Intermediate **I1** (4-chloro-1,2-dimethylquinolin-1-ium iodide) and Intermediate **I2** (2,3-dimethylbenzo[d]thiazol-3-ium iodide) was obtained by the reaction of 4-chloro-2-methylquinoline and 2-methylbenzo[d]thiazole with iodomethane. Then the key intermediate (Z)-1,2-dimethyl-4-((3-methylbenzo[d]thiazol-2(3H)-ylidene)methyl) quinolin-1-ium iodide (**I3**) was prepared by the reaction of **I1** and **I2** by following the reported procedures^27^. The target compounds with different styryl substituents introduced at ortho-position of **I3** were then achieved by the reaction of **I3** with a variety of aromatic aldehydes. The target compounds were obtained with high isolated yields (80–90%) and were characterized by MS and NMR. All the spectral data were in agreement with the proposed structures. The purities of these compounds were confirmed to be above 95% using HPLC analysis.

**Table 1.** List of 3-methylbenzo[d]thiazol-methylquinolinium derivatives (**A1–A16**).


			
			
			
			

### *In vitro* antibacterial activity

The antibacterial activities of the compounds were first evaluated by using a standard two-fold micro-dilution assay in Mueller–Hinton broth against a panel of drug-sensitive bacterial strains, including *B. subtilis* 168, *S. aureus* (ATCC 29213), *E. coli* (ATCC 25922), *E. faecium* (ATCC 49624), *E. faecalis* (ATCC 29212) and *S. epidermidis* (ATCC 12228). Methicillin, vancomycin, and berberine were tested under the same assay conditions as reference compounds. The minimum inhibition concentration (MIC) results were summarized in Table 2. By comparing the results with berberine, all compounds tested exhibit a much superior antibacterial activity against the six selected drug-sensitive strains. It is noteworthy that compounds **A2**, **A3**, and **A5** have the MIC values lower than 6 μg/mL, showing their efficacy is comparable to clinical antibiotics such as methicillin and vancomycin. From Table 2, the structural effects arising from different styryl substituents show strong influence on the antibacterial activity as indicated by their MIC values. It seems that the antibacterial activity of these new compounds is correlated with the size and the styryl substituents (R group). Compound **A2** (para-fluorostyryl) and **A5** (para-methylstyryl) were showing very similar MIC value in the experiment, while other substituents with increase molecular size, particularly **A13**–**A16**, the antibacterial activity were decreased. This is most probably due to the steric influence of the substituent in the binding domain of FtsZ protein.

**Table 2.** Minimum inhibitory concentrations of compounds **A1**–**A16** against a panel of drug-sensitive bacterial strains (*μ*g/mL).

Cpd.	*B. subtilis* 168	*S. aureus* ATCC 29213	*E. faecium* ATCC 49624	*E. faecalis* ATCC 29212	*S. epidermidis* ATCC 12228	*E. coli* ATCC 25922
**A1**	4	4	8	8	4	12
**A2**	1.5	1.5	2	2	1	3
**A3**	2	2	4	4	2	6
**A4**	12	12	16	16	12	32
**A5**	2	2	2	2	2	3
**A6**	2	4	6	6	4	12
**A7**	4	4	8	8	4	12
**A8**	4	4	8	8	4	24
**A9**	8	8	16	16	8	48
**A10**	8	8	12	12	8	16
**A11**	6	6	8	8	6	16
**A12**	8	8	8	8	8	24
**A13**	12	16	16	16	12	32
**A14**	32	32	64	64	32	>64
**A15**	16	16	24	24	16	32
**A16**	24	24	32	32	24	64
Berberine	128	128	>192	>192	96	>192
Methicillin	0.5	1	4	6	0.5	4
Vancomycin	1	1	2	>64	0.5	2

Based on the antibacterial results of the drug-sensitive strains, six compounds with the best MIC values (**A1**–**A3** and **A5**–**A7**) were selected for further evaluation against an extended panel of clinically relevant drug-resistant bacterial strains including MRSA (ATCC BAA41, ATCC 43300), Vancomycin-Resistant *E. faecalis* (ATCC 51575) and *E. faecium* (ATCC 700221), NDM-1 *E. coli* (ATCC BAA2469), *P. aeruginosa* (ATCC BAA2108). As shown in Table 3, the selected compounds exhibited potent activity against three MRSA strains with MIC values of 1.5–4 μg/mL. The MIC values are comparable to that of vancomycin. Among these compounds, **A2** was the most effective with a MIC value of 1.5 μg/mL against MRSA, and showed more than 100-fold better antibacterial activity than that of berberine and methicillin. The growth of vancomycin-resistant *E. Faecalis* and *E. faecium* (VREs) were inhibited with MIC values of 2–8 μg/mL. The antibacterial effects of these quinolinium derivatives on the VREs were much better that of vancomycin and berberine (MICs >64 μg/mL) and were comparable to that of methicillin. When tested on the Gram-negative strains NDM-1 *E. coli* and *P. aeruginosa*, methicillin shows little effect on the gowth of thesesuperbugs even at a high concentration (192 μg/mL). It is noteworthy that **A2** and **A5** can effectively inhibit the growth of NDM-1 *E. coli* with a much lower MIC value of 3 μg/mL. Moreover, *P. aeruginosa* which is resistant to most of the clinical antibiotics can be effectively inhibited by our quinolinium derivatives **A2** and **A5** with the MIC values of 6–8 μg/mL.

**Table 3.** The antibacterial activity of selected compounds against drug-resistant bacterial strains (*μ*g/mL).

Cpd.	*S. aureus* ATCC BAA41^a^	*S. aureus* ATCC 33591^a^	*S. aureus* ATCC 43300^a^	*E. faecalis* ATCC 51575^b^	*E. faecium* ATCC 700221^b^	*E. coli* ATCC BAA2469^c^	*P. aeruginosa* ATCC BAA2108^d^
**A1**	4	4	4	8	8	12	32
**A2**	1.5	1.5	1.5	2	2	3	6
**A3**	2	2	2	4	4	6	24
**A5**	2	2	2	2	2	3	8
**A6**	4	4	4	6	6	12	24
**A7**	4	4	4	8	8	12	32
Berberine	192	192	192	>192	>192	>192	>192
Methicillin	>192	>192	>192	6	6	>192	>192
Vancomycin	2	2	2	>64	>64	6	>64

a Methicillin-resistant *S. aureus*.

b Vancomycin-resistant strains.

c *E. coli* expressing NDM-1 beta-lactamase.

d A multidrug-resistant strain.

### Time-killing curve determinations

**Figure 2.**
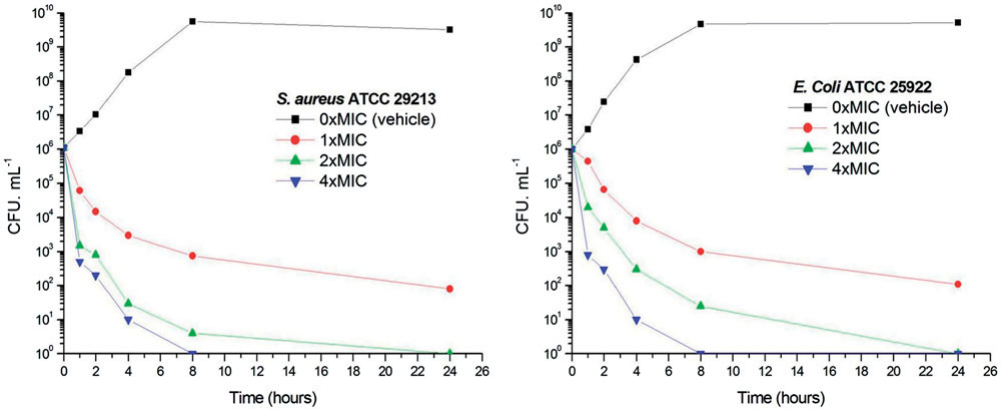
Time-killing curve of **A2**. (A) At time zero, samples of a growing culture of *S. aureus* ATCC 29213 were incubated with concentrations of **A2** equivalent to 1 × (red), 2 × (green), or 4 × (blue) the MIC. (B) Samples of a growing culture of *E. coli* ATCC 25922 were incubated with concentrations of **A2** equivalent to 1 × (red), 2 × (green), or 4 × (blue) the MIC. Vehicle (1% DMSO; black) was included. Samples were removed at the time intervals indicated for the determination of viable cell counts.

To further investigate whether the antibacterial activities of 3-methylbenzo[d]thiazol-methylquinolinium derivatives are bactericidal or not, the viable counts for the determination of killing curves were performed as previous report^25^. Time killing curves resulting from **A2** against *S. aureus* ATCC 29213 and *E. coli* ATCC 25922 are presented in Figure 2. A significant reduction about 10^3^ CFU mL^−1^ (99.9% of bacterial growth inhibited) was observed in 2 h at 2 × and 4 × MIC against *S. aureus*. These phenomena indicate that **A2** can inhibit the growth of *S. aureus* quickly. Figure 2(A) showed that **A2** at 1 × MIC concentration caused a reduction of 1 × 10^2^ CFU mL^−1^ for *S. aureus* in 4 h and is below the lowest detectable limit (10^3^ CFU mL^−1^) in 24 h. In the *E. coli* bacterial survival assay, 4 × MIC of **A2** can rapidly reduce the viable counts below the lowest detectable limit after 2 h incubation, and the counts at MIC concentration were maintained under the lowest detectable limit for over 24 h (Figure 2(B)). These results indicate that quinolinium derivatives can inhibit the bacteria growth quickly through the bactericidal mode.

### Effects of quinolinium derivatives on the GTPase activity of FtsZ

**Figure 3.**
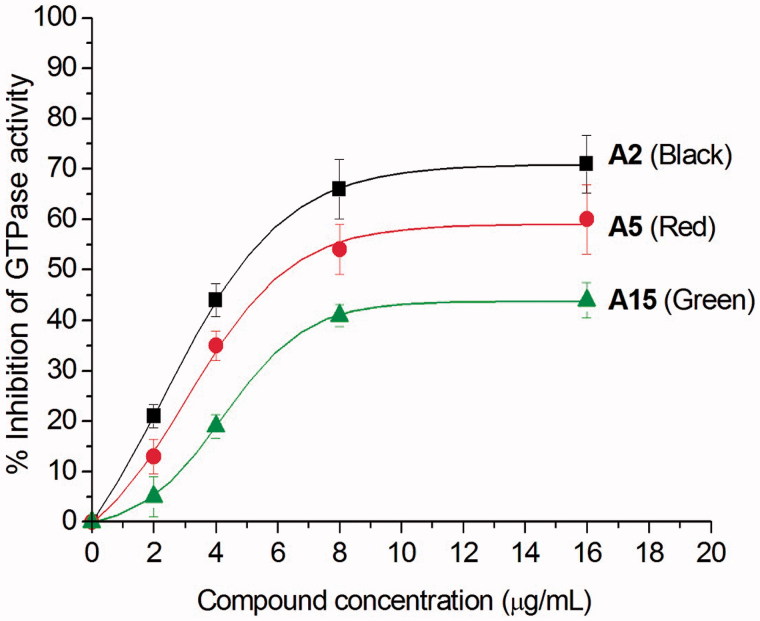
Inhibition of GTPase activity of *Sa*FtsZ by quinolinium derivatives **A2**, **A5** and **A15**.

After examining the antibacterial activity of quinolinium derivatives, we further investigated their mode of action. Recent study reported that the antibacterial activity of zantrin Z3 and quinoline derivative (Figure 1) may due to their interferential effect on the GTPase activity of FtsZ^13–15^. To confirm whether the antibacterial activity of our quinolinium derivatives also follows this mechanism, we studied the effects with three selected compounds (**A2**, **A5,** and **A15**) which possess different antibacterial activities, on the GTPase activity of purified *S. aureus* FtsZ (*Sa*FtsZ). The results shown that the quinolinium derivatives inhibit GTP hydrolysis of FtsZ in a concentration-dependent manner. For example, **A2** at the concentration of 4 μg/mL showed an inhibition of 45% and achieved to 70% inhibitory effect when using 16 μg/mL (Figure 3). Athough **A5** and **A15** being less effective on the inhibition experiments, the results demonstrated that the compounds are able to inhibit the proliferation of bacteria *via* influencing the GTPase activity of FtsZ.

### Effects of quinolinium derivatives on the FtsZ polymerization

**Figure 4.**
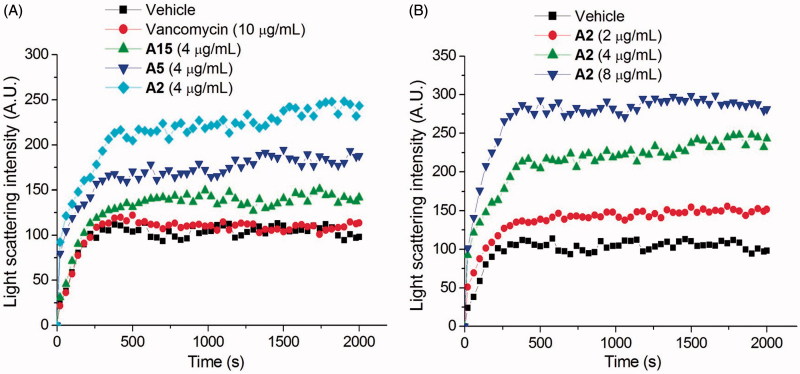
Effect of quinolinium derivatives on the polymerization of FtsZ. (A) Effect on the polymerization of FtsZ in the absence or in the presence of 4 μg/mL of compound **A2**, **A5,** and **A15**, or 10 μg/mL of vancomycin. (B) The polymerizations of FtsZ in the presence of compound **A2** at the concentrations of 2, 4, and 8 μg/mL.

**Figure 5.**
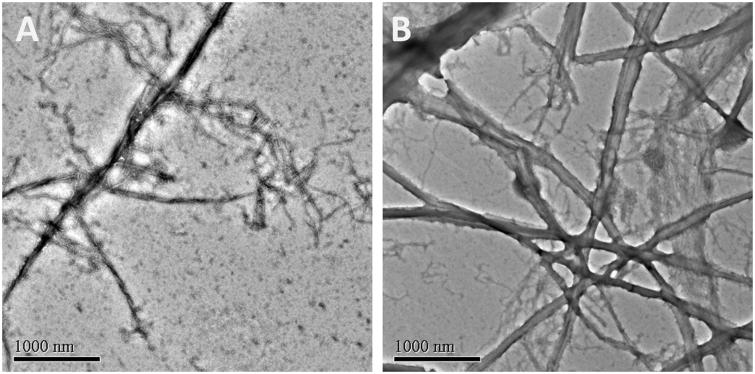
Electron micrographs of FtsZ polymers in the absence (A) and in the presence (B) of 4 μg/mL of **A2**.

Recent studies revealed that the dynamic polymerization of FtsZ was determined by its guanosine triphosphatase activity^7^^,^^8^. In order to understand the mechanism of antibacterial activities of these quinolinium derivatives, we used a light scattering assay to assess the impact of selected compounds on the polymerization dynamic of FtsZ. It was found that the selected compounds (**A2**, **A5**, and **A15**) at 4 μg/mL exhibited an obvious enhancement effect on FtsZ polymerization. Vancomycin (10 μg/mL) is also tested as a non-FtsZ-targeting control antibiotic. As expected, it dose not disrupt the FtsZ polymerization. As shown in Figure 4(A), **A2** possesses a stronger impact than that of **A5** and **A15** on the FtsZ polymerization and this result is correlated with their effects on GTPase activity of FtsZ and the antibacterial activity. Figure 4(B) shows the time-dependent polymerization profiles of *Sa*FtsZ in the absence and presence of **A2** at a concentration range from 2 to 8 μg/mL. The results confirmed that **A2** stimulates FtsZ polymerization in a concentration-dependent manner. The effect of **A2** on FtsZ polymerization was also observed *via* transmission electron microscopy (TEM). It was found that the size of FtsZ polymers were sharply increased after the treatment with **A2** at 4 μg/mL (Figure 5). These phenomena suggested that the inhibition of GTPase activity of FtsZ is attributed to the disruption of FtsZ polymerization dynamic.

### Effects of quinolinium derivatives on the morphology of B. subtilis

**Figure 6.**
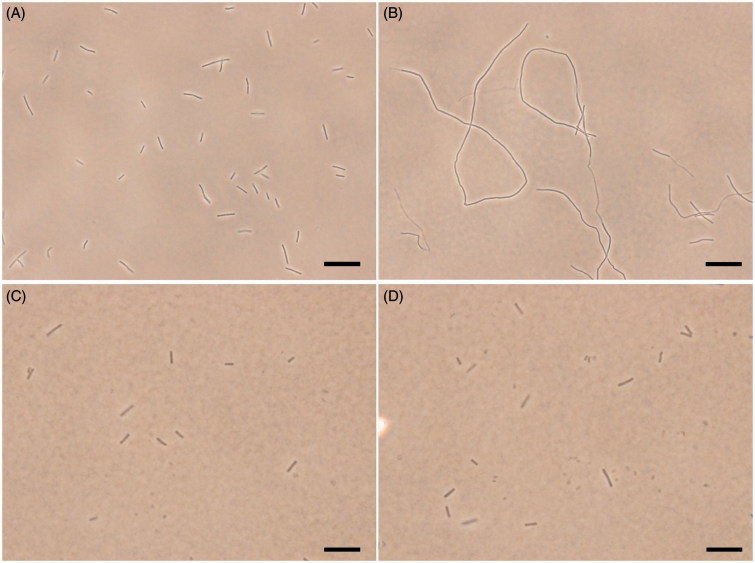
Inhibition of cell division by **A2**. Cells of *B. subtilis* 168 were grown in the absence (A), and presence of **A2** (B), CTAB (C) and methicillin (D) at the MIC concentration. Scale Bar =10 μm.

To further investigate the mechanism of the antibacterial activity of the quinolinium derivatives, we observed the bacterial morphology of *B. subtilis* incubated with/without **A2** through an optical microscopy. In this assay, cetyltrimethylammonium bromide (CTAB, MIC is 1 μg/mL against *B. subtilis*), a membrane-targeting antiseptic agent and methicillin, a cell wall synthesis inhibitor were used as negative controls. Figure 6(A) shows that untreated *B. subtilis* cells have typical short rod morphology with cell lengths from 2 to 10 μm. After treatment with 1.5 μg/mL of **A2**, the cell morphology of *B. subtilis* was found to become long filament. Most of the cell lengths are longer than 20 μm (Figure 6(B)). On the other hand, cells treated with CTAB or methicillin at MIC concentration did not show any filamentation (Figures 6(C,D)), suggesting the antibacterial mechanism of these quinoliniium derivatives is different to that of CTAB and methicillin. It is noteworthy that similar cell elongation phenomena can also be found in other FtsZ inhibitors such as berberine and benzamide derivatives^19^^,^^28–30^. These results strongly indicated that our quinolinium derivatives inhibit cell division through disrupting GTPase activity and dynamic polymerization of FtsZ, then leading to cell death.

### Predicted binding mode of quinolinium derivative (A2) in FtsZ

**Figure 7.**
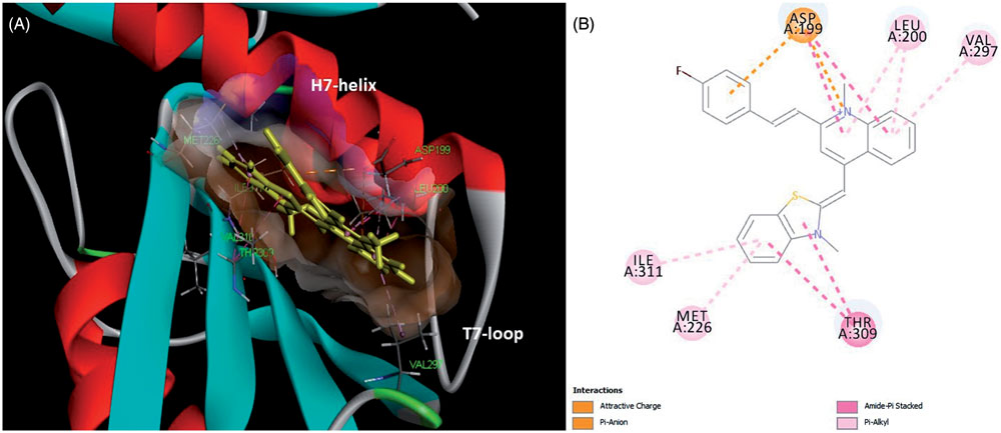
(A) Quinolinium derivative (**A2**) was predited to bind into the C-terminal interdomain cleft of FtsZ; (B) predicted interactions between **A2** and the amino acids of FtsZ.

To study the binding mode of our quinolinium derivatives in the FtsZ protein, molecular modeling was used to identify a potential binding site for these small molecules in the FtsZ. A 2.01 Å crystal structure of *S. aureus* FtsZ apo-form^26^ and **A2** were used as models for this purpose. The highest docking score suggested that the ligand bind near the T7-loop and H7-helix of FtsZ (Figure 7(A)). Since the binding site is a relatively narrow cleft composed by T7-loop, H7-helix and a four-stranded β-sheet, the substrate requires some degree of planarity in its structure to fit in. The docking results suggested that a large number of favorable hydrophobic interactions occur between the molecule and the side chains of Asp199, Leu200, Met226, Ile228, Val297, Thr309 and Ile311. Moreover, the cationic pyridinium of **A2** is predicted to participate in the charge interaction with the negative charged side chain of Asp199 (Figure 7(B)).

### Cytotoxicity of quinolinium derivatives

To probe for any potential mammalian cytotoxicity, the MTS tetrazolium assay was used to assess the cytotoxicity of selected quinolinium derivatives, which possess better antibacterial activity than other derivatives (**A2** and **A5**), against two mammalian cell lines (HK-2, L929). Both tested compounds were found to be minimally toxic to these cell types, with 50% inhibitory concentrations (IC50s) higher than 40 μg/mL (Table 4), which are much higher than their MIC values (1.5 to 3.0 μg/mL) against MRSA and VRE bacterial strains (Table 3), indicating no significant toxicity towards normal mammalian cells.

**Table 4.** Cytotoxicity of **A2** and **A5** on mammalian cells.

Cpd.	IC_50_ against HK-2	IC_50_ against L929
**A2**	78.25 μM (∼43.2 μg/mL)	82.74 μM (∼45.7 μg/mL)
**A5**	73.85 μM (∼40.5 μg/mL)	78.65 μM (∼43.1 μg/mL)

## Conclusion

In conclusion, a series of new quinolinum derivatives were synthesized by systematically varying a styryl substituent at the ortho-position of 1-methylquinolinium core and their *in vitro* antibacterial activities were investigated comprehensively. The results indicate that these compounds possess significant antibacterial activity against the tested pathogens including the drug-resistant strains of MRSA, VRE, and NDM-1 *E. coli*. It is noteworthy that the MIC values of several compounds (**A1**, **A2**, **A3**, **A5**, **A6**, and **A7**) against MRSA strains are range from 1.5 to 4 μg/mL. The MIC values were found comparable to vancomycin and much lower than that of methicillin. In addition, these quinolinum derivatives exhibited potent antibacterial activity against VREs. In particular, **A2** has the MIC values against vancomycin-resistant *E. faecalis* and *E. faecium* of 2 μg/mL, which are significantly lower than the MIC values of vancomycin (MICs >64 μg/mL). Moreover, **A2** and **A5** can effectively inhibit the growth of multidrug-resistant Gram-negative strains with the MIC values lower than 8 μg/mL, which is much lower than that of methicillin (MICs >192 μg/mL). In addition, the cytotoxicty values of **A2** and **A5** are much higher than their MIC values, suggesting these compounds possess little or low toxicity on the mammalian cells. The investigation on the mode of action revealed that the selected compounds can effectively disrupt the GTPase activity and polymerization of FtsZ in a dose-dependent manner. These results suggest that the interaction between quinolinum derivatives and the C-terminal interdomain cleft interferes with the GTPase activity of FtsZ, which in turn disrupts the polymerization of FtsZ, leading to the abnormal bacterial cell division and inhibition of cell proliferation. Therefore, the quinolinum derivatives could be useful in the development of antibacterial agents against drug resistant pathogens.

## References

[1] Wright GD. Antibiotics: a new hope. Chem Biol 2012;19:3–10. 22284349 10.1016/j.chembiol.2011.10.019

[2] Solomon SL, Oliver KB. Antibiotic resistance threats in the united states: stepping back from the brink. Am Fam Physician 2014;89:938–41. 25162160

[3] Willyard C. The drug-resistant bacteria that pose the greatest health threats. Nature 2017;543:15. 28252092 10.1038/nature.2017.21550

[4] Walsh TR, Weeks J, Livermore DM, Toleman MA. Dissemination of ndm-1 positive bacteria in the New Delhi environment and its implications for human health: An environmental point prevalence study. Lancet Infect Dis 2011;11:355–62. 21478057 10.1016/S1473-3099(11)70059-7

[5] Devasahayam G, Scheld WM, Hoffman PS. Newer antibacterial drugs for a new century. Expert Opin Investig Drugs 2010;19:215–34. 10.1517/13543780903505092 PMC2831224 20053150

[6] Lock RL, Harry EJ. Cell-division inhibitors: New insights for future antibiotics. Nat Rev Drug Discov 2008;7:324–38. 18323848 10.1038/nrd2510

[7] Yang X, Lyu Z, Miguel A, et al Gtpase activity-coupled treadmilling of the bacterial tubulin ftsz organizes septal cell wall synthesis. Science 2017;355:744–7. 28209899 10.1126/science.aak9995 PMC5851775

[8] Bisson-Filho AW, Hsu YP, Squyres GR, et al Treadmilling by ftsz filaments drives peptidoglycan synthesis and bacterial cell division. Science 2017;355:739–43. 28209898 10.1126/science.aak9973 PMC5485650

[9] Haeusser DP, Margolin W. Splitsville: Structural and functional insights into the dynamic bacterial z ring. Nat Rev Microbiol 2016;14:305–19. 27040757 10.1038/nrmicro.2016.26 PMC5290750

[10] Hurley KA, Santos TM, Nepomuceno GM, et al Targeting the bacterial division protein ftsz. J Med Chem 2016;59:6975–98. 26756351 10.1021/acs.jmedchem.5b01098 PMC13006086

[11] Haranahalli K, Tong S, Ojima I. Recent advances in the discovery and development of antibacterial agents targeting the cell-division protein ftsz. Bioorg Med Chem 2016;24:6354–69. 27189886 10.1016/j.bmc.2016.05.003 PMC5157688

[12] Li X, Ma S. Advances in the discovery of novel antimicrobials targeting the assembly of bacterial cell division protein ftsz. Eur J Med Chem 2015;95:1–15. 25791674 10.1016/j.ejmech.2015.03.026

[13] Margalit DN, Romberg L, Mets RB, et al Targeting cell division: Small-molecule inhibitors of ftsz gtpase perturb cytokinetic ring assembly and induce bacterial lethality. Proc Natl Acad Sci USA 2004;101:11821–6. 15289600 10.1073/pnas.0404439101 PMC511058

[14] Nepomuceno GM, Chan KM, Huynh V, et al Synthesis and evaluation of quinazolines as inhibitors of the bacterial cell division protein ftsz. ACS Med Chem Lett 2015;6:308–12. 25815151 10.1021/ml500497s PMC4360151

[15] Mathew B, Ross L, Reynolds RC. A novel quinoline derivative that inhibits mycobacterial ftsz. Tuberculosis (Edinb) 2013;93:398–400. 23647650 10.1016/j.tube.2013.04.002 PMC3686551

[16] Kim E, Lee SH, Lee SJ, et al New antibacterial-core structures based on styryl quinolinium. Food Sci Biotechnol 2017;26:521–9. 10.1007/s10068-017-0072-8 PMC6049449 30263574

[17] Chanawanno K, Chantrapromma S, Anantapong T, et al Synthesis, structure and in vitro antibacterial activities of new hybrid disinfectants quaternary ammonium compounds: pyridinium and quinolinium stilbene benzenesulfonates. Eur J Med Chem 2010;45:4199–208. 20619939 10.1016/j.ejmech.2010.06.014

[18] Sun N, Du RL, Zheng YY, et al Antibacterial activity of n-methylbenzofuro[3,2-b]quinoline and n-methylbenzoindolo[3,2-b]-quinoline derivatives and study of their mode of action. Eur J Med Chem 2017;135:1–11. 28426995 10.1016/j.ejmech.2017.04.018

[19] Sun N, Chan F-Y, Lu Y-J, et al Rational design of berberine-based ftsz inhibitors with broad-spectrum antibacterial activity. PLoS ONE 2014;9:e97514. 24824618 10.1371/journal.pone.0097514 PMC4019636

[20] Chan FY, Sun N, Neves MAC, et al Identification of a new class of ftsz inhibitors by structure-based design and in vitro screening. J Chem Inf Model 2013;53:2131–40. 23848971 10.1021/ci400203f

[21] Sun N, Zheng YY, Du RL, et al New application of tiplaxtinin as an effective ftsz-targeting chemotype for an antimicrobial study. Medchemcomm 2017;8:1909–13. 10.1039/c7md00387k PMC6072346 30108711

[22] Chan FY, Sun N, Leung YC, Wong KY Antimicrobial activity of a quinuclidine-based ftsz inhibitor and its synergistic potential with β-lactam antibiotics. J Antibiot 2015;68:253–8. 25293977 10.1038/ja.2014.140

[23] Chan FY, Neves MA, Sun N, et al Validation of the ampc β-lactamase binding site and identification of inhibitors with novel scaffolds. J Chem Inf Model 2012;52:1367–75. 22559726 10.1021/ci300068m

[24] Chan KF, Sun N, Yan SC, et al Efficient synthesis of amine-linked 2,4,6-trisubstituted pyrimidines as a new class of bacterial ftsz inhibitors. ACS Omega 2017;2:7281–92. 10.1021/acsomega.7b00701 PMC6044853 30023544

[25] Wikler MA, Hindler JF, Cookerill FR, et al Methods for dilution antimicrobial sucseptibility tests for bacteria that grow aerobically. Clinical and Laboratory Standards Institute, Wayne, PA, 2009, pp. M07–A08.

[26] Tan CM, Therien AG, Lu J, et al Restoring methicillin-resistant staphylococcus aureus susceptibility to beta-lactam antibiotics. Sci Transl Med 2012;4:126ra135. 10.1126/scitranslmed.3003592 22440737

[27] Lu YJ, Deng Q, Hou JQ, et al Molecular engineering of thiazole orange dye: Change of fluorescent signaling from universal to specific upon binding with nucleic acids in bioassay. ACS Chem Biol 2016;11:1019–29. 26752011 10.1021/acschembio.5b00987

[28] Haydon DJ, Stokes NR, Ure R, et al An inhibitor of ftsz with potent and selective anti-staphylococcal activity. Science 2008;321:1673–5. 18801997 10.1126/science.1159961

[29] Haydon DJ, Bennett JM, Brown D, et al Creating an antibacterial with in vivo efficacy: Synthesis and characterization of potent inhibitors of the bacterial cell division protein ftsz with improved pharmaceutical properties. J Med Chem 2010;53:3927–36. 20426423 10.1021/jm9016366 PMC2874265

[30] Ma S, Cong C, Meng X, et al Synthesis and on-target antibacterial activity of novel 3-elongated arylalkoxybenzamide derivatives as inhibitors of the bacterial cell division protein ftsz. Bioorg Med Chem Lett 2013;23:4076–9. 23770057 10.1016/j.bmcl.2013.05.056

